# Antidepressant efficacy of administering repetitive transcranial magnetic stimulation (rTMS) with psychological and other non-pharmacological methods: a scoping review and meta-analysis

**DOI:** 10.1017/S0033291725000315

**Published:** 2025-02-27

**Authors:** Cristian G. Giron, Alvin H.P. Tang, Minxia Jin, Georg S. Kranz

**Affiliations:** 1 Department of Psychology, The University of Hong Kong, Hong Kong SAR, China; 2 Department of Rehabilitation Sciences, The Hong Kong Polytechnic University, Hong Kong SAR, China; 3 Shanghai YangZhi Rehabilitation Hospital (Shanghai Sunshine Rehabilitation Center), School of Medicine, Tongji University, Shanghai, China; 4 Mental Health Research Center (MHRC), The Hong Kong Polytechnic University, Hong Kong SAR, China

**Keywords:** brain state, depression, meta-analysis, psychological interventions, rTMS, transcranial magnetic stimulation

## Abstract

To optimize the antidepressant efficacy of repetitive transcranial magnetic stimulation (rTMS), it is important to examine the impact of brain state during therapeutic rTMS. Evidence suggests that brain state can modulate the brain’s response to stimulation, potentially diminishing antidepressant efficacy if left uncontrolled or enhancing it with inexpensive psychological or other non-pharmacological methods. Thus, we conducted a PRISMA-ScR-based scoping review to pool studies administering rTMS with psychological and other non-pharmacological methods. PubMed and Web of Science databases were searched from inception to 10 July 2024. Inclusion criteria: neuropsychiatric patients underwent rTMS; studies assessed depressive symptom severity; non-pharmacological tasks or interventions were administered during rTMS, or did not include a wash-out period. Of 8,442 studies, 20 combined rTMS with aerobic exercise, bright light therapy, cognitive training or reactivation, psychotherapy, sleep deprivation, or a psychophysical task. Meta-analyses using random effects models were conducted based on change scores on standardized scales. The effect size was large and therapeutic for uncontrolled pretest-posttest comparisons (17 studies, Hedges’ *g* = −1.91, (standard error) *SE* = 0.45, 95% (confidence interval) *CI* = −2.80 to −1.03, *p* < 0.01); medium when studies compared active combinations with sham rTMS plus active non-pharmacological methods (8 studies, *g* = −0.55, *SE* = 0.14, 95% *CI* = −0.82 to −0.28, *p* < 0.01); and non-significant when active combinations were compared with active rTMS plus sham psychological methods (4 studies, *p* = 0.96). Attempts to administer rTMS with non-pharmacological methods show promise but have not yet outperformed rTMS alone.

## Introduction

Repetitive transcranial magnetic stimulation (rTMS) is a multi-tool for clinical practice and psychological research, with recommended applications across neuropsychiatric conditions (Lefaucheur et al., 2020). As an antidepressant, the efficacy of rTMS is supported by meta-analyses for major depressive disorder (MDD) (Hyde et al., 2022), and across neuropsychiatric conditions when applied over the left dorsolateral prefrontal cortex (Kan et al., 2023). Strategies to improve the efficacy and consistency of rTMS protocols focus on personalizing stimulation targets or parameters that maximize the dose of stimulation (Klooster, Ferguson, Boon, & Baeken, 2022; Padberg et al., 2021). However, what should occur during treatment sessions has received much less attention. What patients do during sessions is typically not reported, and advice is not provided by expert guidelines (Lefaucheur et al., 2020) or cannot be established due to a lack of evidence (Hebel et al., 2022). When details are reported, patients are instructed to relax, e.g., “During 10 Hz rTMS or iTBS sessions, participants were instructed to keep their eyes open and relax” (Bulteau et al., 2022). But expecting patients with depressive syndromes to consistently follow these instructions is problematic: internalizing disorders, such as depressive disorders, are characterized by emotional and cognitive dyscontrol linked to abnormal brain activity and connectivity (Disner, Beevers, Haigh, & Beck, 2011; Goldstein-Piekarski et al., 2022). Since distinct behaviors and thoughts correspond to different brain states (i.e., activity and connectivity), and as different brain states may underlie receptivity to stimulation, it has been suggested that patients’ actions and thoughts during stimulation contribute to the highly variable outcomes of rTMS (Sack et al., 2024; Schutter, Smits, & Klaus, 2023; Silvanto, Muggleton, & Walsh, 2008). Such heterogeneity poses a significant challenge for antidepressant rTMS therapies. For instance, a meta-analysis of gold-standard trials applying rTMS to the left dorsolateral prefrontal cortex (DLPFC) found a large effect size but also substantial heterogeneity, indicated by a Higgin’s I^2^ of 86% (Kan et al., 2023). Further, inconsistent changes in brain hemodynamics are observed in both the targeted prefrontal region and remote areas during and after rTMS (Xia et al., 2024).

### Evidence for the ‘brain state’ hypothesis of rTMS

The potential to use psychological and other non-pharmacological methods to alter the brain’s response to rTMS has been discussed previously in non-systematic reviews and studies with healthy participants. In reviews focusing on the cognitive neuroscience literature, Silvanto and colleagues suggest that TMS does not broadly excite, inhibit, or induce ‘virtual lesions’ of targeted brain regions (Silvanto et al., 2008; Silvanto & Pascual-Leone, 2008). Instead, neuronal pools within these regions respond distinctly to stimulation, depending on their activity during stimulation. Similar reasoning has driven experiments to explore whether behavior during rTMS modulates brain responses in healthy participants. Neuroimaging studies show that the after-effects of high-frequency and high-intensity rTMS over the left dorsal premotor cortex are excitatory when the left hand is actively gripping during stimulation but inhibitory when the left hand is at rest (Bestmann et al., 2008). Similar effects were observed using continuous and intermittent theta burst stimulation protocols (Huang, Rothwell, Edwards, & Chen, 2008). In another study, Luber and colleagues (Luber, Balsam, Nguyen, Gross, & Lisanby, 2007) paired audio-visual stimuli with single pulses in a classical conditioning task. The presentation of conditioned stimuli, but not unconditioned stimuli, before TMS attenuated motor-evoked potentials. More recently, a study interleaving TMS-fMRI found varied blood-oxygenation changes in response to TMS, depending on whether healthy participants were at rest or performing the n-back task (Grosshagauer et al., 2024). These neuroimaging studies with healthy participants support the hypothesis that brain state at the time of TMS modulates the brain response to stimulation. However, behavioral studies do not support this hypothesis (Bakulin et al., 2020; Dalhuisen et al., 2023; Hoy, Enticott, Daskalakis, & Fitzgerald, 2010). To wit, behavioral after-effects following high-frequency rTMS of the left DLPFC are not modulated when healthy participants viewed positive compared to neutral affect-inducing images during stimulation (Hoy et al., 2010), nor when stimulation is delivered during a working memory task compared to rTMS alone (Bakulin et al., 2020). However, these behavioral studies assessed after-effects with task performance or self-reported mood items that may not be sufficiently sensitive. Indeed, meta-analyses of studies with healthy participants report that rTMS alone has null or little effects on these behavioral measures (Remue, Baeken, & De Raedt, 2016; Xu et al., 2024).

### Previous reviews or perspectives on brain states and rTMS

The evidence supports the hypothesis that rTMS after-effects can be influenced by participants’ thoughts or actions during stimulation. This possibility has attracted significant interest from researchers and clinicians, leading to several reviews and perspectives on the topic (Kochanowski, Kageki-Bonnert, Pinkerton, Dougherty, & Chou, 2024; Sack et al., 2024; Sathappan, Luber, & Lisanby, 2019; Schutter et al., 2023; Tatti et al., 2022; Tsagaris, Labar, & Edwards, 2016; Wilkinson, Holtzheimer, Gao, Kirwin, & Price, 2019). However, these have been non-systematic narrative reviews or overviews (Sack et al., 2024; Sathappan et al., 2019; Schutter et al., 2023; Tsagaris et al., 2016; Wilkinson et al., 2019), did not examine treatment efficacy (Tsagaris et al., 2016), or did not consider the timing of the combined interventions (Kochanowski et al., 2024; Sathappan et al., 2019; Tatti et al., 2022; Wilkinson et al., 2019). We determined a scoping review was necessary to charter this literature and estimate antidepressant efficacy, as we expected the literature to be highly heterogeneous that is inherent in rTMS research (Hyde et al., 2022; Kan et al., 2023), compounded by the variability of psychological tasks, interventions, and their timing with rTMS. To our knowledge, this is the first systematic effort to chart and conduct a meta-analysis on this research question. We aim to ground these findings by comparing efficacy estimates with published meta-analyses on the efficacy of rTMS alone and conduct power analyses to guide future research.

### Objectives

This scoping review systematically charts and meta-analyzes the literature on the antidepressant efficacy of rTMS administered during or immediately after non-pharmacological tasks and interventions – putatively, rTMS with brain state manipulated. We included studies that combined these methods to treat any neuropsychiatric condition and reported effects on depression severity. Findings are discussed to inform combination designs for clinical and experimental research. The specific research objectives are as follows:

1. To investigate whether the antidepressant effects of rTMS are modulated (i.e., identify synergistic or antagonistic effects) by combination with psychological tasks and interventions, or by other non-pharmacological methods.
2. To charter the literature relevant to future clinical and research paradigms integrating clinical rTMS protocols with psychological methods.

As this scoping review was conducted to chart the literature systematically, it was not restricted to controlled studies. Controlled conditions are often challenging to implement, as they require multiple comparisons (e.g., active combinations versus sham rTMS, sham psychological method, or sham combinations), or controls for psychological methods are not standardized. Most available efficacy data come from uncontrolled studies because the research to date has been largely exploratory. Therefore, both controlled and uncontrolled meta-analyses are reported and discussed, with careful consideration given to the weight of evidence when making claims or inferences.

## Methods

This scoping review complies with the reporting guidelines of the Preferred Reporting Items for Systematic Reviews and Meta-Analyses extension for Scoping Reviews (PRISMA-ScR) (Tricco et al., 2018). Its protocol was registered with the Open Science Framework on 25 June 2022 (https://osf.io/n8hw3).

### Eligibility criteria

The PICO model summarizing inclusion criteria is as follows:

- *Patient:* Human neuropsychiatric patients with depressive symptoms. Treated patients do not need to have a depressive disorder as a primary diagnosis. For example, if treated patients are diagnosed with post-traumatic stress disorder or a pain disorder, the study can be included if depression severity is assessed.
- *Intervention:* Non-pharmacological intervention overlaps with sessions of rTMS. Chronotherapies can be included if wash-out periods are not used. Combined neuromodulation or open- and closed-loop designs are excluded, as this literature is reviewed elsewhere. Studies are also excluded if only pharmacotherapy is combined with rTMS.
- *Comparison:* No restriction.
- *Outcome:* The study reports depression severity such as with the Hamilton Depression Rating Scale (HDRS), visual analogue scales, or assessments by trained clinicians

Exclusion criteria were: 1) rTMS was administered alone; 2) timing of treatments was not concurrent with rTMS; 3) combined rTMS with pharmacotherapy only; 4) combined rTMS with other neuromodulatory devices or utilized an open- or closed-loop design without a non-pharmacological task or intervention.

### Information sources and search strategy

The search was performed using broad terms for rTMS and depression in the PubMed database until 5 May 2022 (initially procured by YTC, HYCC, SWC, YYC) and updated until 10 July 2024 to also include Web of Science database (by CGG, AHPT, MJ). Search queries used for each database are shown in Supplementary Table S1. Search results were exported into EndNote 20 to facilitate screening. This was done independently by separate reviewer teams (initially conducted by YTC, HYCC and SWC, YYC), then updated by a separate team (by CGG and AHPT, MJ). The reference lists of relevant reviews and included studies were also screened. Screening involved multiple teams due to the intentionally broad search queries and inclusion criteria. Furthermore, reporting what patients do during rTMS sessions is unstandardized in this literature. As a result, many studies were expected to require careful full-text screening to determine whether non-pharmacological methods were combined with rTMS and, if so, whether they were administered concurrently.

### Study selection and data chartering

Reviewers first screened by title and abstract independently, then discussed and merged these screening results. Full-text retrieval and screening were then conducted by the same independent teams. Disagreements on study selection or inclusion were resolved by discussion and consensus with GSK. No automation tools were used for full-text screening. A customized Excel spreadsheet was developed for data chartering by CGG (items are described in the next section). Once the full-text screening was completed, data was extracted by CGG, YTC, HYCC, SWC, and YYC, then verified by MJ and AHPT.

### Extracted data

The following items were extracted using a customized Excel spreadsheet: participant characteristics for each treatment group (diagnoses and diagnostic method; age; sex ratio); rTMS protocol (target site, pulse pattern, and intensity, number of sessions); psychological task or intervention; timing of treatments; measurements of antidepressant outcome; reported results of statistical comparisons; estimated effect sizes for within-subjects and between-subjects comparisons for each study. If these data were visually presented but not numerically available, we used WebPlotDigitizer to extract numerical values (https://automeris.io/WebPlotDigitizer/, last accessed 23 June 2023). If information was not obtainable in the full-text or Supplementary Material of a study, corresponding authors were emailed.

### Groups and comparisons

To assess antidepressant efficacy, change scores were calculated for each group across treatment timepoints by subtracting baseline scores from endpoint scores on standardized clinical scales, such as the HDRS. These change scores were then pooled with respect to comparison. Effect sizes were estimated for within-group comparisons (posttest minus pretest) and controlled comparisons (change scores of the active intervention minus control condition). We estimated separate meta-analytic effect sizes for the following comparisons, where PSYC refers to psychological or non-pharmacological task or intervention:

- Uncontrolled: [active rTMS + active PSYC] endpoint versus baseline scores.
- Controlled: [active rTMS + active PSYC] versus [active rTMS + sham PSYC].
- Controlled: [active rTMS + active PSYC] versus [sham rTMS + active PSYC].
- Controlled: [active rTMS + active PSYC] versus [sham rTMS + sham PSYC].

These comparisons are necessary tests for the hypothesis that combining treatments alters efficacy, as they serve as controls for both rTMS and PSYC.

For studies where statistical comparisons were not reported (e.g., case studies), post-treatment response or remission outcomes were extracted to estimate the antidepressant efficacy. Such studies were not included in any meta-analysis.

### Effect sizes based on change scores of within-group and between-group comparisons

To compute effect sizes, we used custom scripts written in Python (version 3.11.5) with NumPy and Panda libraries in a Jupyter Notebook environment. Standardized mean differences were based on change scores of individual study comparisons. Details of relevant algorithms (Hedges, 1981; Higgins et al., 2019) and pseudocode is provided in Supplementary Text 1.

### Meta-analysis and tests of heterogeneity

Meta-analyses were conducted when at least three included studies conducted similar comparisons. We used the *metafor* package (Viechtbauer, 2010) in R version 4.3.3 (R Core Team, 2024) to conduct the meta-analyses and meta-regressions, to assess publication and sensitivity bias, and to conduct subgroup analyses described below. Random-effect models were used for all comparisons to account for predicted variability among study effects. The Restricted Maximum Likelihood (REML) method was used to estimate the between-study variance.

To assess heterogeneity, we used Cochran’s Q test and Higgin’s I^2^ statistic. A significant Q test indicates the presence of heterogeneity among study effects beyond sampling error. The *I^2^* statistic is used to estimate the proportion of total variation due to heterogeneity rather than sampling error: *I^2^* values below 30% indicate low heterogeneity, between 30–60% indicate moderate heterogeneity and values above 60% indicate substantial heterogeneity (Higgins et al., 2019).

### Publication and sensitivity bias analyses

Publication bias was assessed using funnel plots and Egger’s regression intercept test in meta-analyses with ten or more studies due to concerns about insufficient power (Sterne et al., 2011). Leave-one-out cross-validation sensitivity tests were conducted to identify whether any single study disproportionately influences meta-analytic results. To assess the robustness of each meta-analytic estimate, we report the range of Hedges’ g’s observed, highlight whether the direction of effect changes, and whether the meta-analytic estimate remains significant.

### Meta-regression

To assess potential moderators, meta-regressions were conducted for meta-analytic estimates consisting of 10 or more studies (Higgins et al., 2019). Moderators were sex ratios (number of male patients divided by the number of female patients in a sample), mean age of a sample, and whether the psychological task or intervention was a supported intervention for depressive symptoms (e.g., psychotherapies) or not (e.g., psychophysical tasks).

### Secondary meta-analysis

Meta-analyses were repeated to include only studies targeting the left DLPFC and recruited patients with depressive disorders. This would allow us to compare meta-analytic findings with a recent cross-diagnostic meta-analysis comparing active rTMS alone to sham rTMS alone (Kan et al., 2023).

### Monte-Carlo simulation-based power analysis

Monte-Carlo simulation-based power-analyses were performed to estimate the sample size needed to obtain sufficient power to investigate the hypothesis that combining rTMS with PSYC is a superior treatment (compared to sham conditions). Methods are described in Supplementary Text 4.

## Results

### Selection and sources of evidence

**Figure 1.**
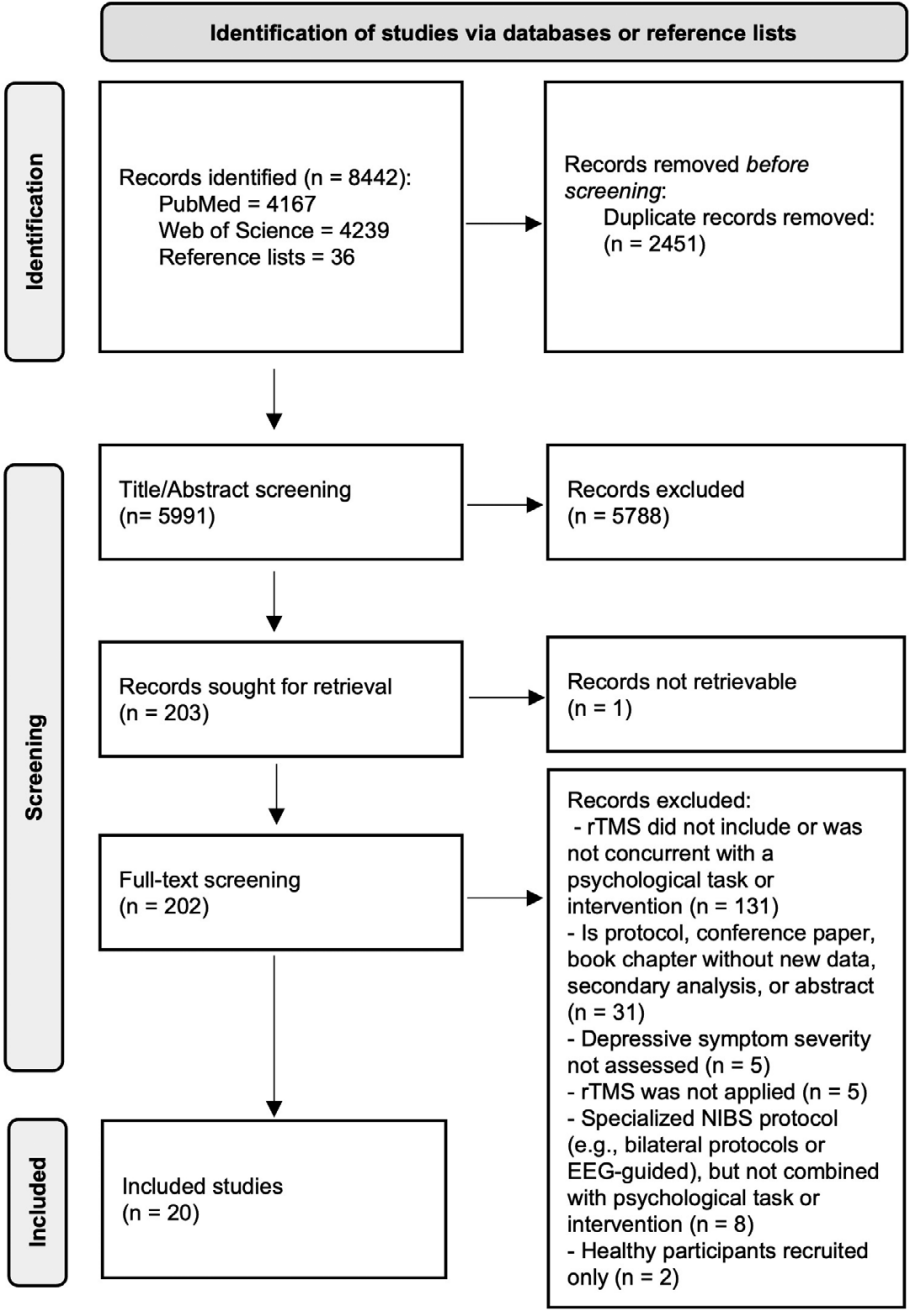
Summary flow-diagram of literature screening.

8442 records were identified in PubMed, Web of Science, and the reference lists of included studies and relevant reviews (Figure 1). The PRISMA-ScR checklist is provided in Supplementary Table S2. References and reasons for 183 excluded studies that underwent full-text screening are shown in Supplementary Table S3. Twenty studies were eligible for inclusion.

### Characteristics of included studies

The literature on modulating antidepressant outcomes of rTMS or psychological interventions by their combination comprised 20 studies (Table 1).

**Table 1.** Summary of included studies and key data characteristics.

Study	Psychological task or intervention (PSYC)	Sample information		rTMS protocol		Timing of PSYC	Assessment and comparison outcomes
Barbini et al. (2021)	Bright Light Therapy (BLT)	Diagnosis: MDD (DSM–5) BD (MINI) [rTMS + BLT] n (Male/Female): 40 (13/27) Age (mean ± SD): 58.8 ± 10.47 [rTMS alone] n (Male/Female): 40 (28/12) Age: 57.72 ± 9.89		Target site: left DLPFC Frequency: n.m. Total no. of rTMS sessions: 15		30 min upon awakening at an individualized time, followed by rTMS at noon	HDRS [rTMS + BLT] *vs* [rTMS alone]: Hedges’ *g* = −1.018 (*SE* = 0.236), ↑ [rTMS + BLT]: *g* = −0.382 (*SE* = 0.209), ↑*
Bentwich et al. (2011)	Cognitive Training (COG)	Diagnosis: Probable early or moderate Alzheimer’s Disease (DSM-IV) 6/8 with comorbidity of depression (n.m.) [rTMS + COG] n: 8 (6/2) Age: 75.4 ± 4.4		Target sites: - Broca & Wernicke - right DLPFC & left DLPFC - R-pSAC & L-pSAC Frequency: 10 Hz Intensity: Broca, right DLPFC & left DLPFC: 90% MT Wernicke, R-pSAC & L-pSAC: 110% MT Pulses per session: 1,200 Total no. of rTMS sessions: 30 sessions (at 5 per week for 6 weeks) + 24 maintenance sessions (2 per week for 3 months)		Interleaved COG between rTMS trains	HDRS [rTMS + COG] after 30 sessions: *g* = −0.999 (*SE* = 0.401); n.s.* [rTMS + COG] after 30 sessions plus 24 maintenance (54 total sessions): *g* = −0.330 (*SE* = 0.340); n.s.*
Caloc’h et al. (2023)	Computerized COG	Diagnosis: post-TBI syndrome (n.m.) Age: 48 (1/0)		Target sites: right and left DLPFC, Broca’s and Wernicke’s language areas, right and left posterior parietal areas Frequency: 10 Hz Intensity: 100% RMT Pulses per session: 2400 Total no. of rTMS sessions: 30		Interleaved COG between rTMS trains	Hospital Anxiety and Depression Scale (HADS) Pre to post intervention score = 18/42 to 30/42
Cavallero et al. (2021)	Mindfulness-Based Cognitive Therapy (MBCT): Audio-guided meditation exercises	Group in 1^st^ site n: 16 (3/13)	Diagnosis: Non-psychotic MDD (n.m.) Age: 48.4 ± 15.2	Target site: Left DLPFC Frequency: 10 Hz Intensity: 120% RMT Pulses per session: min: 3,000	Total no. of rTMS sessions: Average: 35 ± 7.7	During rTMS	IDS-SR [rTMS + MBCT]: *g* = −2.050 (*SE* = 0.421), ↑*
		Group in 2^nd^ site n: 11 (5/6)		Target sites: right DLPFC → left DLPFC Frequency: 1 Hz (right DLPFC) → 20 Hz (left DLPFC) Intensity: 120% RMT Pulses per session: 360 (right DLPFC) → 1,200 (left DLPFC)			
Donse, Padberg, Sack, Rush, and Arns (2018)	Psychotherapy: Consisted of evidence-based methods, e.g., Cognitive Behavioral Therapy (CBT), schema therapy, eye movement desensitization and reprocessing, schema therapy	[HF rTMS + psychotherapy] n: 74 (n.m.) [LF rTMS + psychotherapy] n: 115 (n.m.)	Diagnosis: Non-psychotic MDD/Dysthymia (MINI/DSM-IV/DSM–5) Age: 43.2 ± 12.9	Target site: left DLPFC rTMS parameters: 10 Hz Intensity: 110–120% RMT Pulse per session: 1,500	Duration per session: 45 mins (rTMS being 20 min, with psychotherapy continuing until 45 minutes) Total no. of rTMS sessions: Average: 20.9 (SD = 7.5) Range: 10–50 sessions	During rTMS & continued after rTMS for 25 mins	BDI [HF rTMS + psychotherapy]: *g* = −1.716 (*SE* = 0.182), ↑* [LF rTMS + psychotherapy]: *g* = −1.428 (*SE* = 0.132), ↑*
				Target site: right DLPFC Frequency: 1 Hz Intensity: 110–120% RMT Pulses per session: 1,200			
				Target site: right DLPFC ➔ left DLPFC Frequency: LF protocol (shorter duration of 1,000 pulses per session)➔ HF protocol at full-length			
Duan et al. (2023)	Mindfulness-Based Stress Reduction (MBSR)	Diagnosis: Post-stroke depression (DSM–5) [active rTMS + active adjunct] n: 23 (19/4) Age: 58.3 ± 13.1 [sham rTMS + active adjunct] n: 24 (20/4) Age: 53.6 ± 13.0 [sham rTMS + sham adjunct] n: 24 (19/5) Age: 54.42 ± 14.27		Target site: left DLPFC Frequency: 10 Hz Intensity: 80% MT Pulses per session: 1,400 Total no. of rTMS sessions: 20		Mindfulness-guided audio during rTMS. Other exercises of MBSR conducted separately from rTMS sessions	HDRS [active rTMS + MBSR] *vs* [sham rTMS + MBSR]: *g* = −0.120 (*SE* = 0.287), ↑ [active rTMS + MBSR] *vs* [sham rTMS + general psychological care]: *g* = −0.213 (*SE* = 0.288), ↑ [active rTMS + MBSR]: *g* = −0.382 (*SE* = 0.209), ↑*
Eichhammer et al. (2002)	Partial Sleep Deprivation (PSD)	Diagnosis: MDD or BD (DSM-IV) [active rTMS + PSD] n: 10 (4/6) Age: 44.9 ± 12.3 [sham rTMS + PSD] n: 10 (1/9) Age: 48.5 ± 11.6		Target site: left DLPFC Frequency: n.m. Intensity: 80% MT Pulses per session: 1,000 Total no. of rTMS sessions: 4		PSD occurred on the night before rTMS	HDRS [active rTMS + PSD] *vs* [sham rTMS + PSD]: day 1 *g* = −0.177 (*SE* = 0.429), n.s.; Day 2–4: ↑ [active rTMS + PSD]: day 1 *g* = −3.064 (*SE* = 0.744), ↑*
Fryml et al. (2019)	Prolonged Exposure (PE) Therapy	Diagnosis: PTSD (DSM–5) [active rTMS + PE] n: 5 (5/0) Age: 27 ± 2.1 [sham rTMS + PE] n: 3 (2/1) Age: 30 ± 2.6		Target site: Left DLPFC or right DLPFC Frequency: 10 Hz Intensity: 120% MT Pulses per session: 6,000 Total no. of rTMS sessions: 8		40 minutes of PE: 5 minutes before rTMS, 30 minutes overlapping with rTMS, and continuing 5 minutes after rTMS	HDRS [active rTMS + PE] *vs* [sham rTMS + PE]: *g* = −1.093 (*SE* = 0.691), ↑ [active rTMS + PE]: ↑*
Isserles et al. (2011)	Cognitive-Emotional Reactivation: positive, negative, or rTMS alone	Diagnosis: non-psychotic MDD (n.m.), TRD [positive + rTMS] n: 14 (7/7) Age: 45.93 ± 12.9 [negative + rTMS] n: 11 (6/5) Age: 41.75 ± 12.7 [rTMS alone] n: 20 (11/9) Age: 45.93 ± 12.98		Target site: left DLPFC Frequency: 20 Hz Intensity: 120% RMT Pulses per session: 1,680 Total no. of rTMS sessions: 20		Before & During rTMS	HDRS [rTMS + positive] *vs* [rTMS alone]: *g* = +0.425 (*SE* = 0.344), n.s. [rTMS + negative] *vs* [rTMS alone]: *g* = +0.805 (*SE* = 0.344), n.s. [rTMS + positive]: *g* = −1.594 (*SE* = 0.392), ↑* [rTMS + negative]: *g* = −1.317 (*SE* = 0.395), ↑*
Kreuzer et al. (2012)	Sleep Deprivation	Diagnosis: acute depressive episode (DSM-IV) [active rTMS + sleep deprivation] n: 21 (10/11) Age: 45.3 ± 8.0 [sham rTMS + sleep deprivation] n: 16 (8/8) Age: 39.9 ± 13.3		Target site: left DLPFC rTMS parameters: 10 Hz Intensity: 110% RMT Pulses per session: 1,000 Total no. of rTMS sessions: 4		Sleep deprivation occurred on the night before rTMS	HDRS [active rTMS + sleep deprivation] *vs* [sham rTMS + sleep deprivation]: day 1: *g* = −0.227 (*SE* = 0.326), n.s. Day 2–4: n.s. [active rTMS + sleep deprivation]: day 1: *g* = −2.226 (*SE* = 0.402), ↑*
Li et al. (2016)	Computerized rACC-Engaging Cognitive Task (RECT)	Diagnosis: MDD (n.m.) [active rTMS + active RECT] n: 12 (4/8) Age: 43.4 ± 9.0 [sham rTMS + active RECT] n: 12 (6/6) Age: 42.4 ± 12.5 [active rTMS + sham RECT]: 12 (5/7) Age: 39.4 ± 13.2		Target site: left DLPFC Frequency:10 Hz Intensity: 100% MT Pulses per session: 1,600 Total no. of rTMS sessions: 10		Immediately before rTMS	HDRS [active rTMS + active RECT] *vs* [sham rTMS + active RECT]: *g* = −1.247 (*SE* = 0.433), ↑ [active rTMS + active RECT] *vs* [active rTMS + sham RECT]: *g* = −0.273 (*SE* = 0.396), ↑ [active rTMS + sham RECT] *vs* [sham rTMS + active RECT]: *g* = −0.928 (*SE* = 0.416), ↑ [active rTMS + active RECT]: *g* = −1.251 (*SE* = 0.371), ↑*
Mania and Kaur (2019)	BLT	Patient Diagnosis (diagnostic method): MDD (n.m.), TRD [rTMS + BLT] n: 6 (n.m.) Age: n.m.		Target site: left DLPFC Frequency: n.m. Intensity: n.m. Pulses per session: n.m. Total no. of rTMS sessions: n.m.		During deep TMS	HDRS [rTMS + BLT]: *g* = −3.967 (*SE* = 1.196), no statistical comparison was reported
Martinotti et al. (2022)	Cue-Induced Craving and Suppression	Diagnosis: Cocaine Use Disorder (DSM–5) [active rTMS+ cue-induced craving] n: 36 (31/5) Age: 35.8 ± 8 [sham rTMS + cue-induced craving] n: 33 (29/4) Age: 37.9 ± 6.6		Target site: left DLPFC Frequency:15 Hz Intensity: 100% RMT Pulses per session: 2400 Total no. of rTMS sessions: 20		During the first 2 minutes of each rTMS session.	MADRS [active rTMS+ cue-induced craving] vs [sham rTMS + cue-induced craving]: *g* = −0.585 (*SE* = 0.255), ↑ [Active rTMS+ cue-induced craving]: *g* = −1.029 (*SE* = 0.203), ↑*
Neacsiu et al. (2018)	Self-System Therapy	Diagnosis: MDD (MINI 6.0), TRD [rTMS + self-system therapy] n: 5 (3/2) Age: 53.8 ± 4.32		Target site: left DLPFC Frequency: 10 Hz Intensity: 120% RMT Pulses per session: 3,000 Total no. of rTMS sessions: 20		From the start of rTMS until after rTMS for approximately 2.5 mins.	HDRS [rTMS + self-system therapy]: *g* = −2.490 (*SE* = 0.865), ↑*
Osuch et al. (2009)	Imaginal Exposure Therapy	Diagnosis: PTSD (n.m.), MDD [active rTMS + exposure] n: 9 (1/8) Age: 41.4 ± 12.3		Target site: right DLPFC Frequency: 1 Hz Intensity: 100% MT Pulses per session: 1,800 Total no. of rTMS sessions: 20		During rTMS	HDRS [active rTMS + exposure] *vs* [sham rTMS + exposure]: *g* = +0.435 (*SE* = 1.063), n.s. [Active rTMS + exposure]: *g* = +0.470 (*SE* = 1.142), n.s.*
Ross, VanDerwerker, George, and Gregory (2023)	Aerobic Exercise	Diagnosis: post-stroke depression (DSM-IV) [active rTMS + aerobic exercise] n: 6 (3/3) Age: 62.3 ± 12.5 [rTMS alone] n: 6 (2/4) Age: 58.3 ± 12.5 [aerobic exercise alone] n: 4 (1/3) Age: 51.5 ± 18.4		Target site: left DLPFC frequency: 10 Hz Intensity: 120% RMT Pulses per session: 5000 Total no. of rTMS sessions: 24		Aerobic exercises occurred within 30 minutes before or after rTMS sessions	PHQ–9 [active rTMS + aerobic exercise]: *g* = −0.859 (*SE* = 1.269), ↑* [active rTMS + aerobic exercise] *vs.* [rTMS alone]: *g* = +0.683 (*SE* = 0.594) [active rTMS + aerobic exercise] *vs.* [aerobic exercise alone]: *g* = −0.578 (*SE* = 0.658)
Russo, Tirrell, Busch, and Carpenter (2018)	Behavioral Activation	Diagnosis: MDD (n.m.), TRD [rTMS + behavioral activation] n: 11 (0/11) Age: 50 ± 18.9		Target site: left DLPFC. Frequency: 10 Hz Intensity: 120% MT Pulses per session: 3000 Total no. of rTMS sessions: mean (SD): 33.0 ± 5.7		Goal attainment before rTMS; encouraged goal planning during stimulation; selection of the next goal after rTMS.	IDS-SR [rTMS + behavioural activation]: *g* = −1.957 (*SE* = 0.501), ↑*
Thierree et al. (2022)	Trauma Script Exposure	Diagnosis: PTSD (DSM-IV) [HF rTMS + trauma script exposure] n: 18 (5/13) Age: 31.3 ± 10.0 [LF rTMS + trauma script exposure] n: 20 (8/12) Age: 33.5 ± 11.1		rTMS parameters: 10 Hz Intensity: 110% RMT	Target site: right DLPFC Pulses per session: 3,000 Total no. of rTMS sessions: 8	During rTMS	HDRS [HF rTMS + trauma script exposure]: *g* = −1.190 (*SE* = 0.300), ↑* [LF rTMS + trauma script exposure]: *g* = −0.593 (*SE* = 0.234), ↑* HF vs LF: n.s.
				rTMS parameters: 1 Hz Intensity: 70% RMT			
VanDerwerker et al. (2018)	Aerobic Exercise	Diagnosis: post-stroke depression (DSM-IV) [active rTMS + aerobic exercise] n: 3 (2/1) Age: 68.0 ± 12.5		Target site: left DLPFC frequency: 10 Hz Intensity: 120% RMT Pulses per session: 5000 Total no. of rTMS sessions: 24		Aerobic exercise occurred within 30 minutes before or after rTMS sessions	PHQ–9 [active rTMS + aerobic exercise]: *g* = −1.508 (*SE* = 0.594), no statistical comparison reported
Vedeniapin, Cheng, and George (2010)	CBT	Diagnosis: MDD (n.m.), TRD [rTMS + CBT] n: 1 (0/1) Age: 26		Target site: left PFC rTMS parameters: 10 Hz Intensity: 120% MT Pulses per session: 6,000 Total no. of rTMS sessions: 39		CBT was delivered in 14/39 rTMS sessions, starting right before rTMS and continuing during rTMS	BDI Patient remitted

↑: Significantly higher efficacy in experimental group [active rTMS + active adjunct] than control group (either sham or active comparator); ↑*: Significant improvement within experimental group; ↓: Significantly lower efficacy in experimental group than control group; >: Significant higher efficacy in [active positive adjunct] than [active negative adjunct]; Hedges’ g is computed from change scores between baseline and immediate assessment after the end of rTMS treatment course; AD: Alzheimer’s disease; BD: Bipolar disorder; BDI: Beck Depression Inventory; CBT: Cognitive Behavioral Therapy; DSM: Diagnostic and Statistical Manual of Mental Disorders; HDRS: Hamilton Depression Rating Scale; HF: high frequency; IDS-SR: Inventory for Depressive Symptomatology (Self-Report); DLPFC: dorsolateral prefrontal cortex; LF: low frequency; L-pSAC: Left-parietal somatosensory association cortex; MADRS: Montgomery-Asberg Depression Rating Scale; MDD: major depressive disorder; MINI: Mini International Neuropsychiatric Interview; n: Sample size; n.m.: Not mentioned; n.s.: non-significant changed or difference between groups comparison; n.s.*: non-significantly change or difference within groups comparison; PHQ-9: Patient Health Questionnaire-9; PTSD: post-traumatic stress disorder; R-pSAC: right-parietal somatosensory association cortex; SD: Standard deviation; SE: standard error; TRD: treatment-resistant depression.

**Figure 2.**
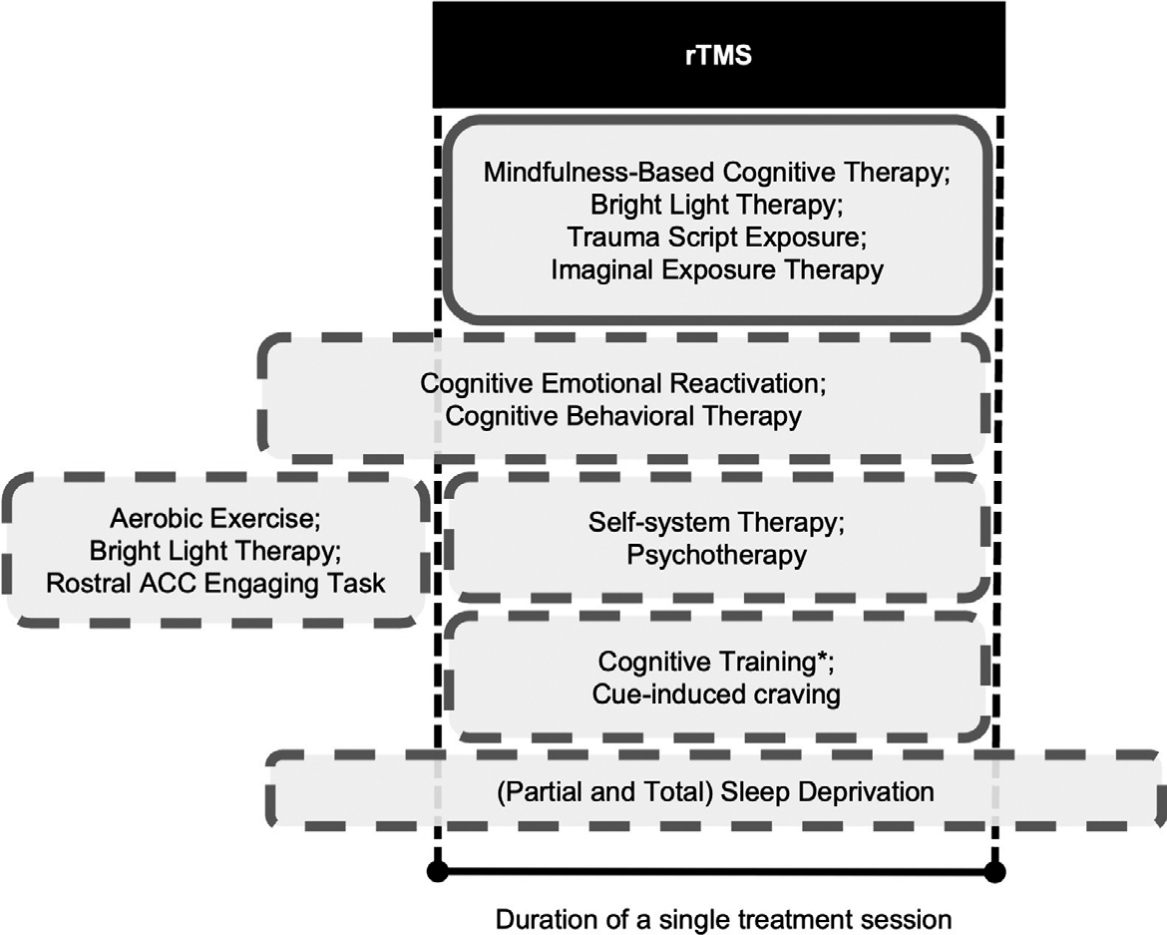
Boxes indicate schedules of non-pharmacological methods that have been attempted before or during sessions of rTMS. For example, the first box below “rTMS” indicates complete concurrence; the next box indicates interventions that start before rTMS and then continue through an rTMS session. Box dimensions are not scaled to durations or any property of the non-pharmacological methods (e.g., dose). *Cognitive training was interleaved with rTMS trains. Abbreviations: rTMS, repetitive transcranial magnetic stimulation; ACC, anterior cingulate cortex.

Thirteen of 20 studies recruited patients diagnosed with MDD, post-stroke depression, or persistent depressive disorder. Six other studies recruited patients with other neuropsychiatric conditions with comorbid depressive symptoms, namely, Alzheimer’s disease, cocaine use disorder, and post-traumatic stress disorder (Table 1). Schedules of non-pharmacological methods combined with rTMS are visualized in Figure 2. Thirteen of 20 included studies reported stabilized pharmacological therapies throughout the study (Supplementary Table S4).

The DLPFC was a prime target, with 17 of 20 studies targeting this region, of which 16 targeted the left DLPFC, two studies targeted the right DLPFC, and four studies targeted bilateral DLPFC (Table 1).

### Meta-analysis results of depression change scores across disorders

The number of included studies allowed for three meta-analyses (out of four discussed in the *Groups and Comparisons* section): within-group comparisons of [active rTMS + active PSYC], between-group comparisons of [active rTMS + active PSYC] versus [active rTMS + sham PSYC]; and between-group comparisons of [active rTMS + active PSYC] versus [sham rTMS + active PSYC].

**Figure 3.**
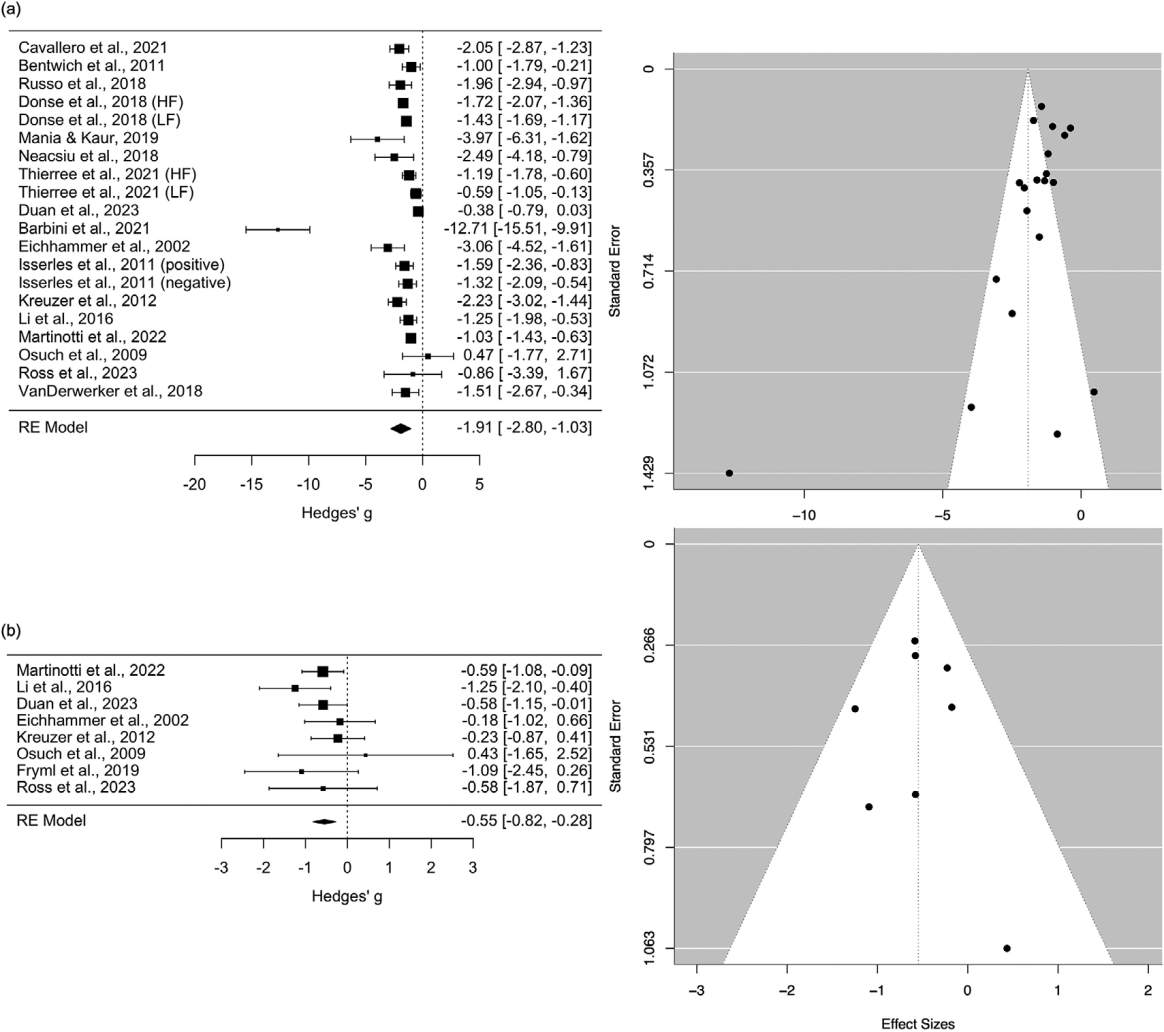
(a) Pooled within-group comparisons (endpoint - baseline) of [active rTMS + active PSYC] treatment. (b) Between-group comparisons of change scores between [active rTMS + active PSYC] and [sham rTMS + active PSYC] groups. Black box sizes indicate weight in the pooled estimate.

Seventeen studies reported endpoint versus baseline changes for [active rTMS + active PSYC]. This uncontrolled effect on depression severity across disorders was large and significantly therapeutic with Hedges’ *g* = −1.91, standard error (*SE*) = 0.45, 95% confidence interval (*CI*) = −2.80 to −1.03), *p* < 0.01). However, these results were substantially heterogeneous (*Q*(*df* = 19) = 126.59, *p* < 0.01, *I^2^* = 97.27%). Meta-regression results suggest age and sex ratio were non-significant moderators (*p* > 0.05), but whether PSYC was an antidepressant intervention was significant (coefficient = −1.745, *SE* = 0.819, 95% *CI* = −3.350 to −0.140, *p* = 0.03). This significant negative moderation indicates that depression severity decreased further when rTMS was combined with a recognized intervention. Leave-one-out sensitivity tests indicated a robust meta-analytic estimate, with *g* ranging between −2.01 to −1.41 (*p* < 0.01 for each iteration). Despite Egger’s test being non-significant, the funnel plot appears asymmetric with a bias toward lower efficacy (Figure 3a).

Eight studies compared [active rTMS + active PSYC] versus [sham rTMS + active PSYC]. The effect size, *g*, was -0.55 (*SE* = 0.14, 95% *CI* = −0.82 to −0.28, *p* < 0.01). These results were not heterogenous (*Q*(*df* = 7) = 5.84, *p* = 0.56, *I^2^* = 0.00%). Leave-one-out sensitivity tests indicated a robust meta-analytic estimate, with *g* ranging between −0.62 to −0.47 (*p* < 0.01 for each iteration). Publication bias did not appear to be an issue (Figure 3b).

Four studies compared groups receiving [active rTMS + active PSYC] versus [active rTMS + sham PSYC]. This controlled effect on depression severity was not significant (Supplementary Text 2).

### Secondary meta-analysis: left DLPFC treatment for depressive disorders

**Figure 4.**
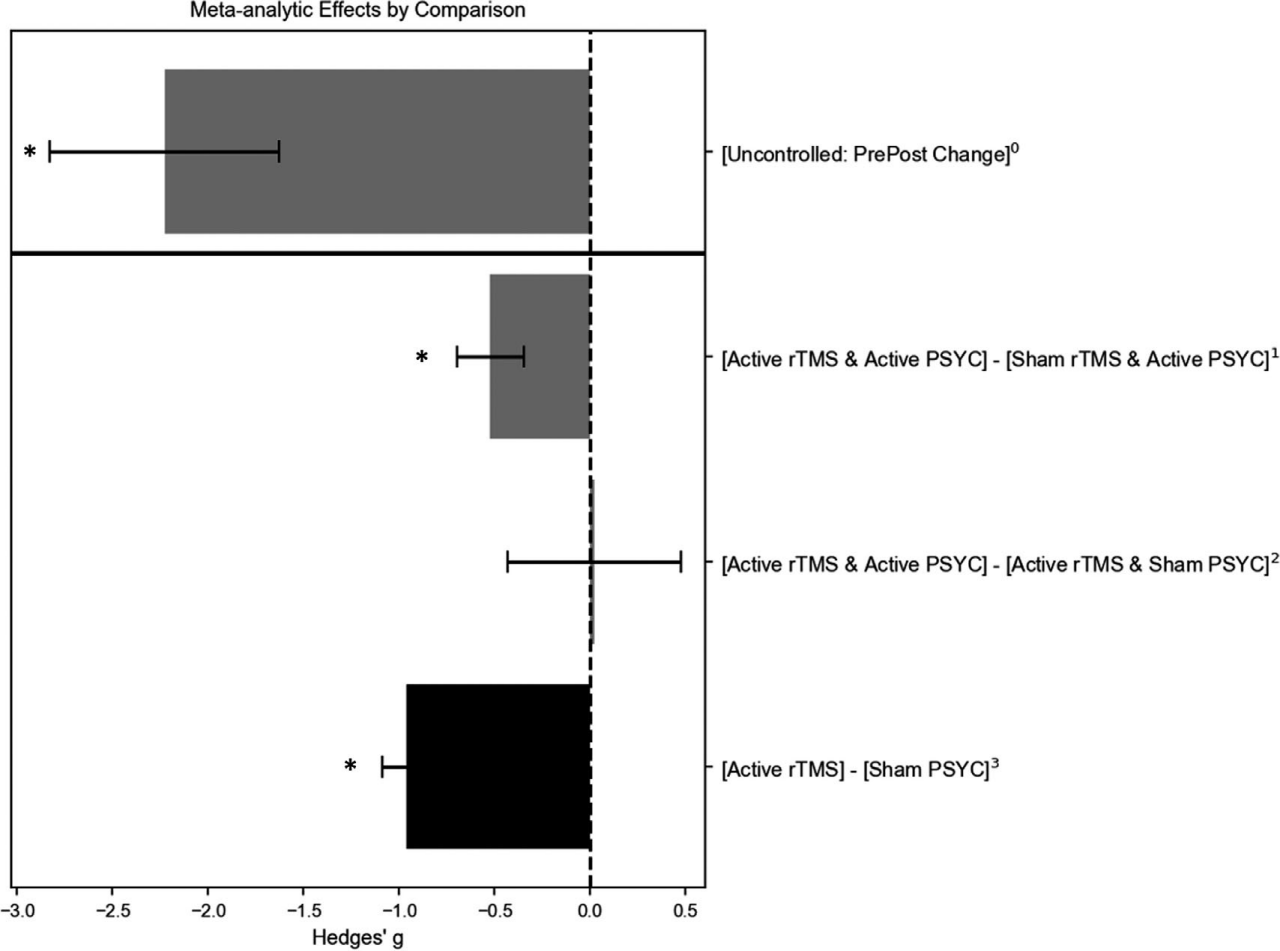
Meta-analytic estimates of studies treating patients with depressive disorders and targeting the left dorsolateral prefrontal cortex (DLPFC). Grey bars are based on findings in this review; the black bar is based on findings from Kan et al. (2023). ^0^Estimate is based on 14 uncontrolled studies (16 datasets); ^1^estimate is based on five controlled trials; ^2^estimate is based on four controlled trials; ^3^estimate is based on 61 controlled trials. * Pooled estimate was statistically significant (*p < 0.01*). PSYC refers to psychological or other non-pharmacological methodss.

The above meta-analyses were repeated but only included studies that targeted the left DLPFC to treat patients with depressive disorders (e.g., MDD or post-stroke depression). Forest and funnel plots are provided in Supplementary Text 3. Meta-analytic estimates are shown in Figure 4.

Within-group comparisons of [active rTMS + active PSYC] at endpoint minus baseline change scores included 14 studies (16 datasets), with a large and significant therapeutic *g* of −2.23 (*SE* = 0.60, 95% *CI* = −3.41 to −1.05, *p* < 0.01), albeit with substantial heterogeneity (*Q*(*df* = 15) = 111.21, *p* < 0.01, *I^2^* = 97.74%). Mixed findings for publication bias were observed: Egger’s test was not significant, but the funnel plot was asymmetric. Leave-one-out sensitivity tests suggested robust findings (*g*-range = −2.39 to −1.59, *p* < 0.01 for each iteration). Age, sex ratio, and whether PSYC was an antidepressant therapy were non-significant moderators.

Five studies compared [active rTMS + active PSYC] versus [sham rTMS + active PSYC] and targeted the left DLPFC of patients with depressive conditions. The effect was medium and significantly therapeutic with a *g* of −0.52 (*SE* = 0.18, 95% *CI* = −0.87 to −0.18, *p* < 0.01). The results were not heterogenous (*Q*(df = 4) = 4.31, *p* = 0.36, *I^2^* = 4.11%). However, leave-one-out sensitivity tests indicated an unstable meta-analytic estimate, with *g* ranging from −0.64 to −0.39 (only non-significant when Duan et al., (2023) was excluded). Publication bias may not be an issue as the funnel plot appeared symmetric.

The four studies that compared [active rTMS + active PSYC] versus [active rTMS + sham PSYC] groups all applied rTMS to the left DLPFC of patients with depressive disorders.

### Power analysis results

Power analysis curves and methods are included in Supplementary Text 4. To test the hypothesis that [active rTMS + active PSYC] is significantly better than [sham rTMS + active PSYC] and [sham rTMS + sham PSYC] but not compared to [active rTMS + sham PSYC], a clinical trial with four independent groups would need to recruit 80 patients (20 patients per group). For the hypothesis that both active treatments are better than all active and sham comparators (including [active rTMS + sham PSYC]), the number of patients needed jumps to 240 (60 patients per group). Only two included studies (Barbini et al., 2021; Duan et al., 2023) were sufficiently powered.

## Discussion

This scoping review reports meta-analytic estimates of the efficacy of combining rTMS with psychological tasks and interventions or other non-pharmacological methods. Results suggest that while the antidepressant efficacy of combining rTMS with psychological methods is promising, it does not surpass the efficacy of rTMS alone. As of 10 July 2024, this literature comprised 20 studies (Table 1) combining rTMS with psychotherapy, bright light therapy, aerobic exercise, partial and total sleep deprivation, cognitive training, cognitive emotional reactivation, and a psychophysical task.

These findings complement a growing body of work, as numerous systematic reviews and meta-analyses have investigated the antidepressant efficacy of pharmacological, psychological, and non-invasive brain stimulation therapies. For example, an umbrella review of meta-analyses found a small yet significant effect size when pharmacological or psychological treatments are used independently (Leichsenring, Steinert, Rabung, & Ioannidis, 2022). The efficacy of rTMS alone also shows promise, with a meta-analysis of various rTMS protocols indicating small to medium effect sizes on depressive conditions (Hyde et al., 2022). In contrast, a cross-diagnostic meta-analysis focusing on protocols targeting the left DLPFC found a medium to large effect size for depressive symptoms across neuropsychiatric conditions (Kan et al., 2023). There is also an increasing interest in identifying synergies between treatments to advance these approaches. For instance, Rakesh, Cordero, Khanal, Himelhoch, and Rush (2024) recently conducted a meta-analysis on the synergistic effects of rTMS with pharmacological treatments, observing a large effect size of rTMS with antidepressants compared to sham rTMS with antidepressants.

### Uncontrolled meta-analysis results

Uncontrolled clinical trials show a large, significant, and robust short-term reduction of depressive symptom severity, measured by comparing treatment endpoint to baseline scores. However, these results are substantially heterogenous with possible publication bias. Findings were consistent when restricted to studies targeting the left DLPFC for treating depressive conditions. Meta-regressions showed that age and sex were not significant moderators for both pooled estimates. We further assessed whether the antidepressant efficacy of rTMS was influenced by the type of psychological method combined with stimulation – specifically, whether the psychological method was a recognized intervention (e.g., psychotherapy) or not (e.g., psychophysical tasks). This analysis was limited to uncontrolled studies comparing endpoint versus baseline change due to the small number of studies. The moderating effects were significant when pooling all studies but were non-significant when restricting the analysis to protocols targeting the left DLPFC. These mixed findings may be due to chance, as pooled estimates showed significant and substantial heterogeneity. Another possibility is that the high efficacy of left DLPFC protocols for treating depressive symptoms overshadowed the antidepressant effects of psychological augmentations (Figure 4). While the large effect sizes observed in the uncontrolled meta-analysis are promising and warrant further investigation, these estimates were uncontrolled and exhibit considerable heterogeneity.

### Controlled meta-analysis results

Control groups are essential baselines for distinguishing combination treatment effects from confounding variables like placebo effects and natural symptom fluctuations in episodic conditions such as depression. Additionally, to draw causal conclusions about synergistic, antagonistic, or null effects of psychological method combinations with rTMS, antidepressant outcomes must be compared with rTMS alone and non-pharmacological methods alone. For example, if combining rTMS with a psychological intervention results in a moderate effect size, but rTMS alone produces a large effect, the outcome might be misinterpreted as synergistic when it is antagonistic. Such antagonistic effects may explain findings by Isserles et al. (2011). An additional concern for rTMS is that its antidepressant effects are substantially heterogeneous (Kan et al., 2023; Klooster et al., 2022; Padberg et al., 2021), making drawing conclusions from uncontrolled observations highly problematic. However, there are challenges with a control condition for psychological interventions, such that few studies could have included sham conditions.

Given these challenges, it was possible to pool study findings for two types of comparisons. In the first comparison, pooling results from seven studies, the active combination treatment [active rTMS + active PSYC] was significantly more effective than [sham rTMS + active PSYC], with a medium effect size. This robust meta-analytic estimate was consistent and supported by a funnel plot indicating no publication bias. In the second comparison, pooling results from three studies, the effect size for [active rTMS + active PSYC] versus [active rTMS + sham PSYC] was non-significant and highly heterogeneous. These results suggest that combining psychological methods with rTMS enhances efficacy, but the reverse effect is not supported. However, it must be emphasized that the number of pooled studies was small for both comparisons, e.g., multiple analyses requiring more than 10 or more pooled studies were not recommended.

The results of various meta-analytic estimates are summarized in Figure 3a and highlighted by a power analysis (Supplementary Text 4). The meta-analytic effect size of active rTMS compared to sham rTMS in patients with depressive conditions is significantly large, though notably heterogeneous (Kan et al., 2023). Relative to this effect size, the benefits of including a psychological task or intervention to enhance the efficacy of rTMS are not supported. The simulations-based power analysis highlights this difference in effect size. Using the observed effect sizes of [active rTMS + active PSYC] versus rTMS alone and assuming significant superiority of combination over rTMS alone, the number of patients needed to obtain sufficient power is 80 patients per group (240 total). Compared with 20 patients per group (80 total) if simply testing the hypothesis that the combination treatment is better than sham rTMS or the active psychological method alone.

### Notable observations from included studies

Bright light therapy with rTMS is the most promising combination therapy for depression. Two controlled studies reported encouraging outcomes: a large effect size indicated superior efficacy when bright light therapy was administered on mornings of rTMS treatment days and compared to rTMS alone (Barbini et al., 2021); and a separate research team observed large reductions in HDRS scores in six patients with treatment-resistant depression when bright light therapy was administered during rTMS sessions (Mania & Kaur, 2019).

This scoping review was motivated by concerns that brain states may influence the antidepressant effects of rTMS. Two identified studies illustrate these concerns through controlled designs. During rTMS, Isserles et al. (2011) guided patients toward negative or positive thinking during rTMS, operationalized as thoughts that mitigate or exacerbate depressive symptoms. Treatment outcomes for these groups were compared to patients receiving rTMS alone. Assessed using Beck’s Depression Index, the authors identified a significant antagonistic effect of the negative thinking condition. These findings need to be replicated as the sample size was small, the finding was not observed when assessing symptom severity with the HDRS, and the design of Isserles et al. (2011) may not dissociate whether negative thinking blocked the effects of rTMS (a ‘brain state’ effect) or reinforced depressive symptoms. Nevertheless, the authors cautioned the need to control for brain state during antidepressant protocols of rTMS (Isserles et al., 2011). In contrast, Li et al. (2016) had patients complete a shape-discrimination task just before high-frequency rTMS, as this task was observed to induce frontal electroencephalogram theta activity (Min & Park, 2010), which persisted after the completion of the psychological task (Li et al., 2016). This combination significantly boosted antidepressant effects compared to [active rTMS + sham PSYC] (with a small effect size; Table 1).

## Limitations

Our screening protocol by title and abstract may have missed studies if these details were only provided in the main text and not the title or abstract or if they were not discussed in relevant reviews (Sathappan et al., 2019; Tatti et al., 2022; Tsagaris et al., 2016; Wilkinson et al., 2019) or not in the reference list of fully screened studies. Meta-analyses for controlled studies were also based on a small number of studies, restricting our analysis to pooled statistics and funnel plots only; Egger’s tests or meta-regressions when the study count was below 10 were not conducted due to the limited power of such analyses. While the primary aim of this review was to identify psychological or other non-pharmacological methods that may modulate brain states and, in turn, influence the antidepressant response to rTMS, we intentionally did not restrict inclusion to a single domain (e.g., cognitive tasks alone). Although it would be ideal to examine these domains independently, the current number of studies is insufficient to support such an analysis.

While our primary analysis adopts a transdiagnostic approach to evaluate the antidepressant efficacy of combination treatments, we must recognize the potential limitations of pooling studies across heterogeneous conditions. Some included conditions, such as PTSD, which may differ substantially from MDD in terms of underlying brain states and etiology (Wang et al., 2024). In response to these concerns, we conducted an additional meta-analysis focusing exclusively on studies of MDD, which is included in Supplementary Text 5. However, a transdiagnostic meta-analysis is presented, as it aligns with recent meta-analytic methods (Begemann, Brand, Curcic-Blake, Aleman, & Sommer, 2020; Kan et al., 2023) and recommendations in mental health care to evaluate treatment efficacy based on symptoms rather than categorical diagnoses (Borsboom, 2017; Kotov et al., 2017; Leucht, van Os, Jager, & Davis, 2024). Future research should further explore how diagnostic differences may influence the outcomes of combination treatments.

Lastly, we attempted to evaluate whether combining rTMS with non-pharmacological methods (e.g., cognitive tasks) leads to different antidepressant efficacy compared to combining rTMS with recognized interventions (e.g., psychotherapy). Meta-regression analyses were only possible for uncontrolled studies that compared changes from baseline to endpoint. The results were mixed: significant moderating effects were found when all studies were pooled but not when analyses were restricted to studies targeting the left DLPFC. More studies are needed to determine whether administering rTMS during non-pharmacological tasks (that optimally control for brain state) yields greater efficacy compared to combining rTMS with established interventions such as psychotherapy. Additionally, studies should investigate the optimal paradigms for such combinations, focusing on distinct domains such as behavioral, cognitive, or emotional functions.

## Conclusion

Attempts to combine rTMS with psychological or other non-pharmacological methods show promise but have yet to surpass the efficacy of rTMS alone. However, this conclusion is based on a limited number of studies, many of which lack rigorous controls (e.g., sham conditions for both rTMS and non-pharmacological interventions). More well-controlled designs are needed to identify combinations that optimize therapeutic outcomes and investigate the underlying mechanisms, such as the role of brain states in shaping these outcomes.

## Supporting information

Giron et al. supplementary material Giron et al. supplementary material

## Supplementary material

To view supplementary material for this article, please visit http://doi.org/10.1017/S0033291725000315.
