# Supplementary figures and images for: Antidepressant efficacy of administering repetitive transcranial magnetic stimulation (rTMS) with psychological and other non-pharmacological methods: a scoping review and meta-analysis
Source: Psychol Med. 2025 Feb 27;55:e64. doi: 10.1017/S0033291725000315 (PMC12080665; doi:10.1017/S0033291725000315)

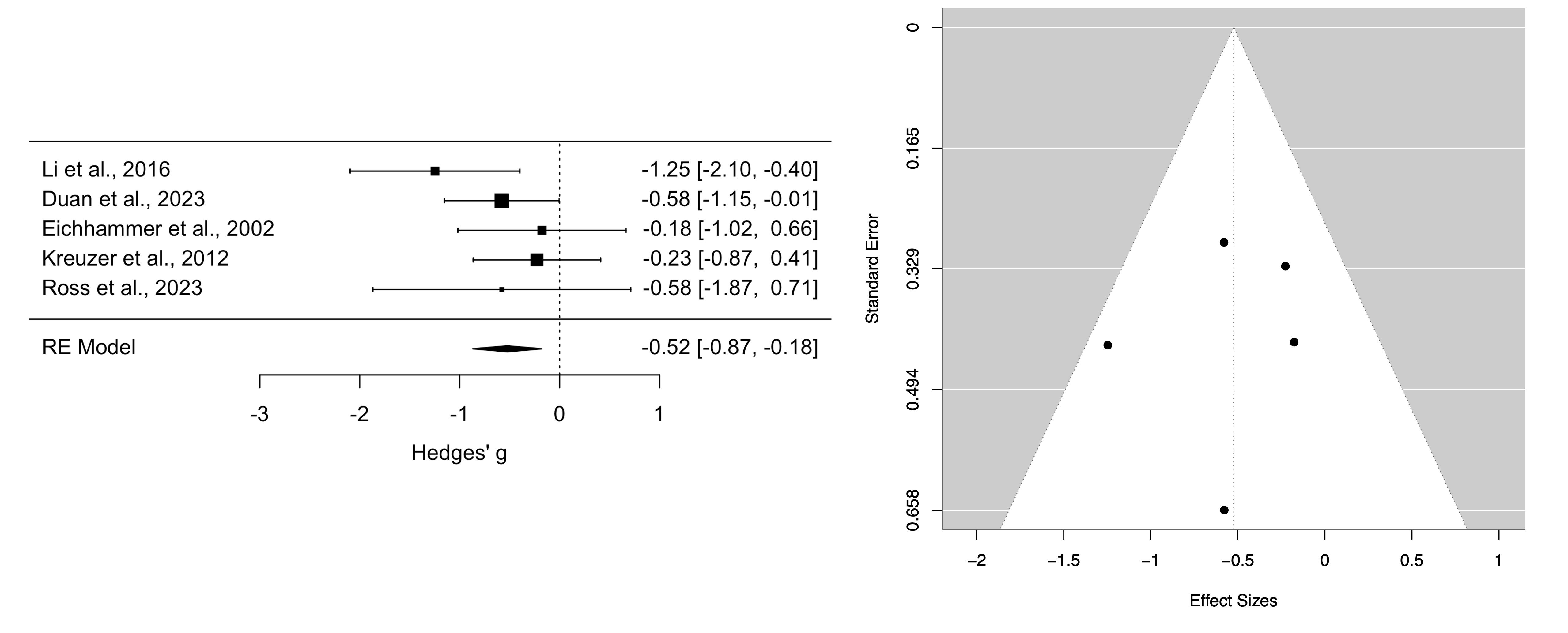

Supplement: Giron et al. supplementary material [file S0033291725000315sup001.zip › Supplementary Figure 3.jpg]

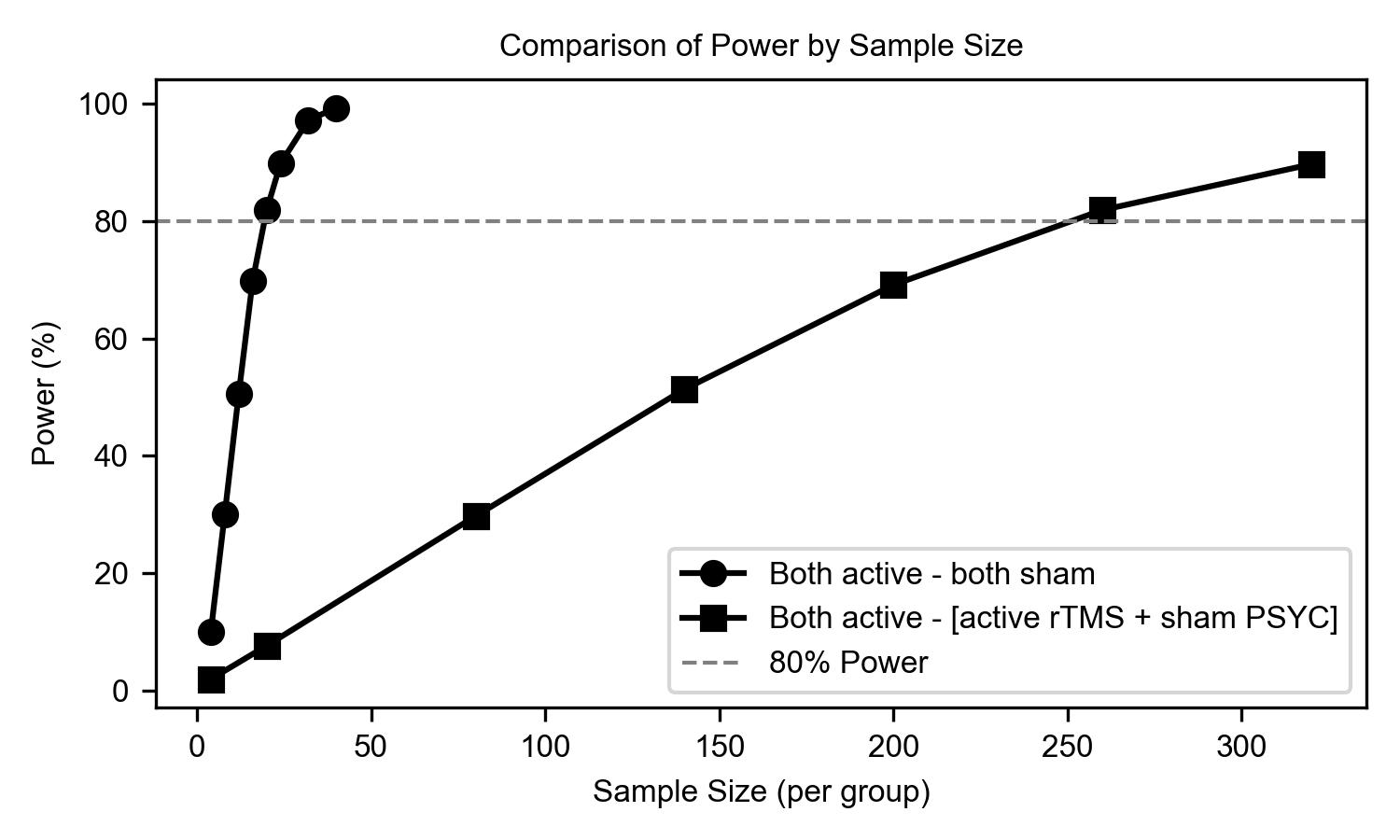

Supplement: Giron et al. supplementary material [file S0033291725000315sup001.zip › Supplementary Figure 4.jpeg]

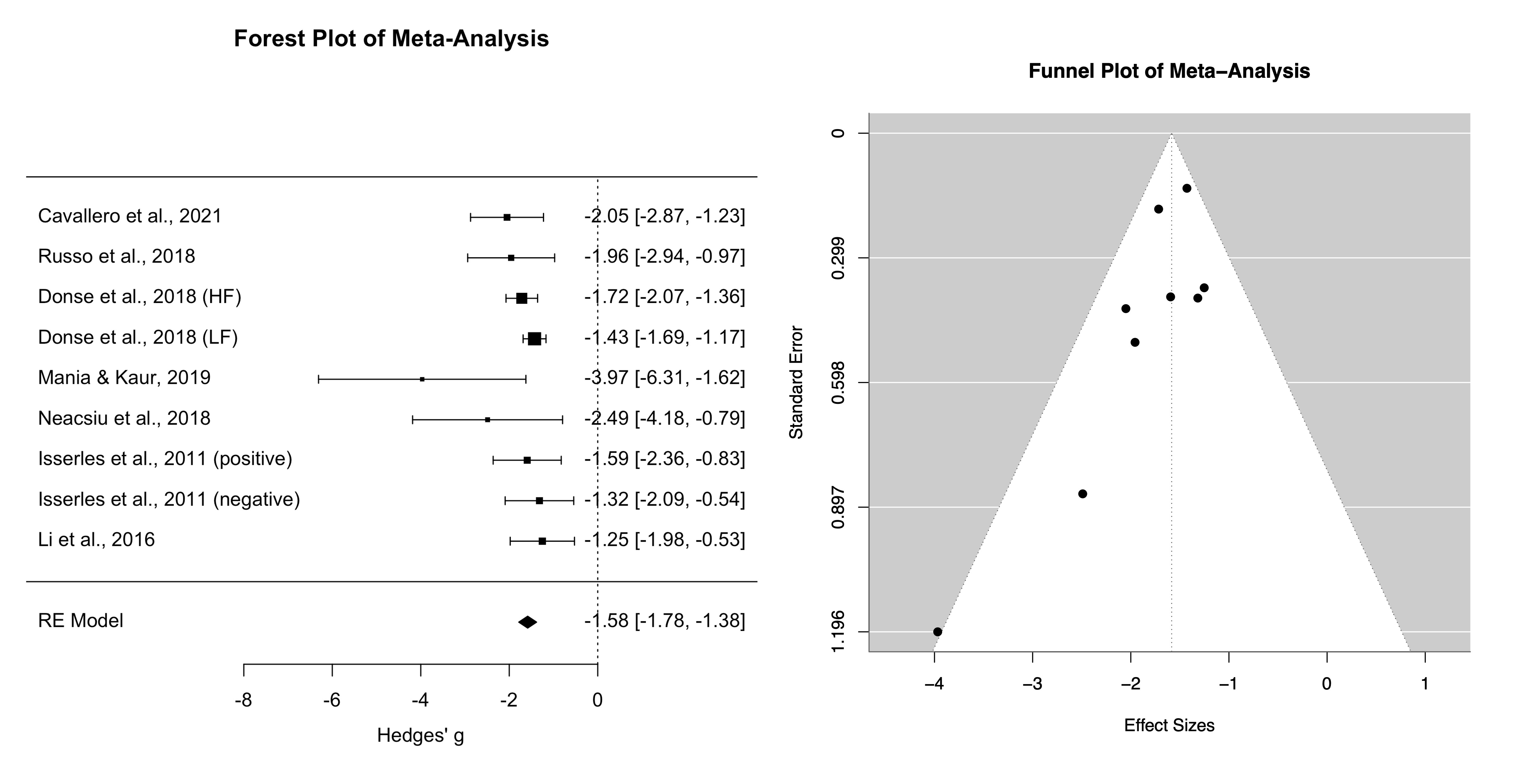

Supplement: Giron et al. supplementary material [file S0033291725000315sup001.zip › Supplementary Figure 5.jpg]

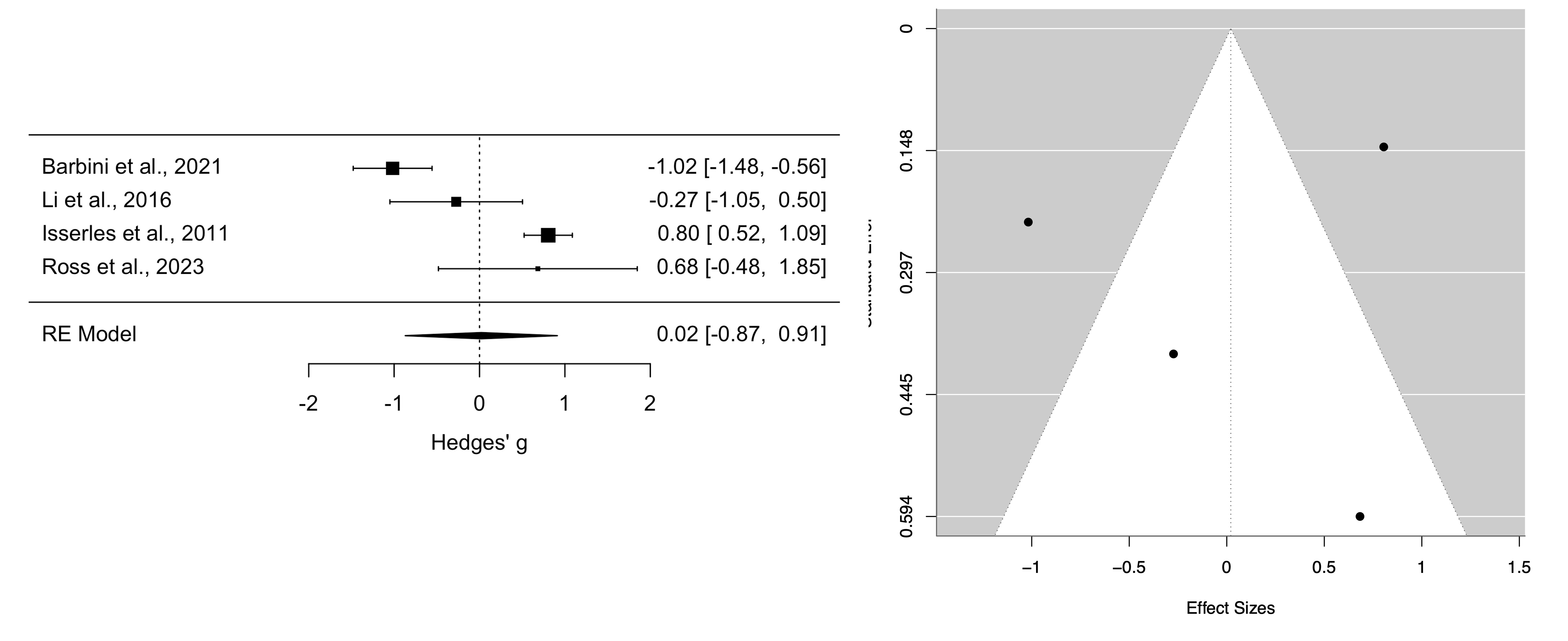

Supplement: Giron et al. supplementary material [file S0033291725000315sup001.zip › Supplementary Figure 1.jpg]

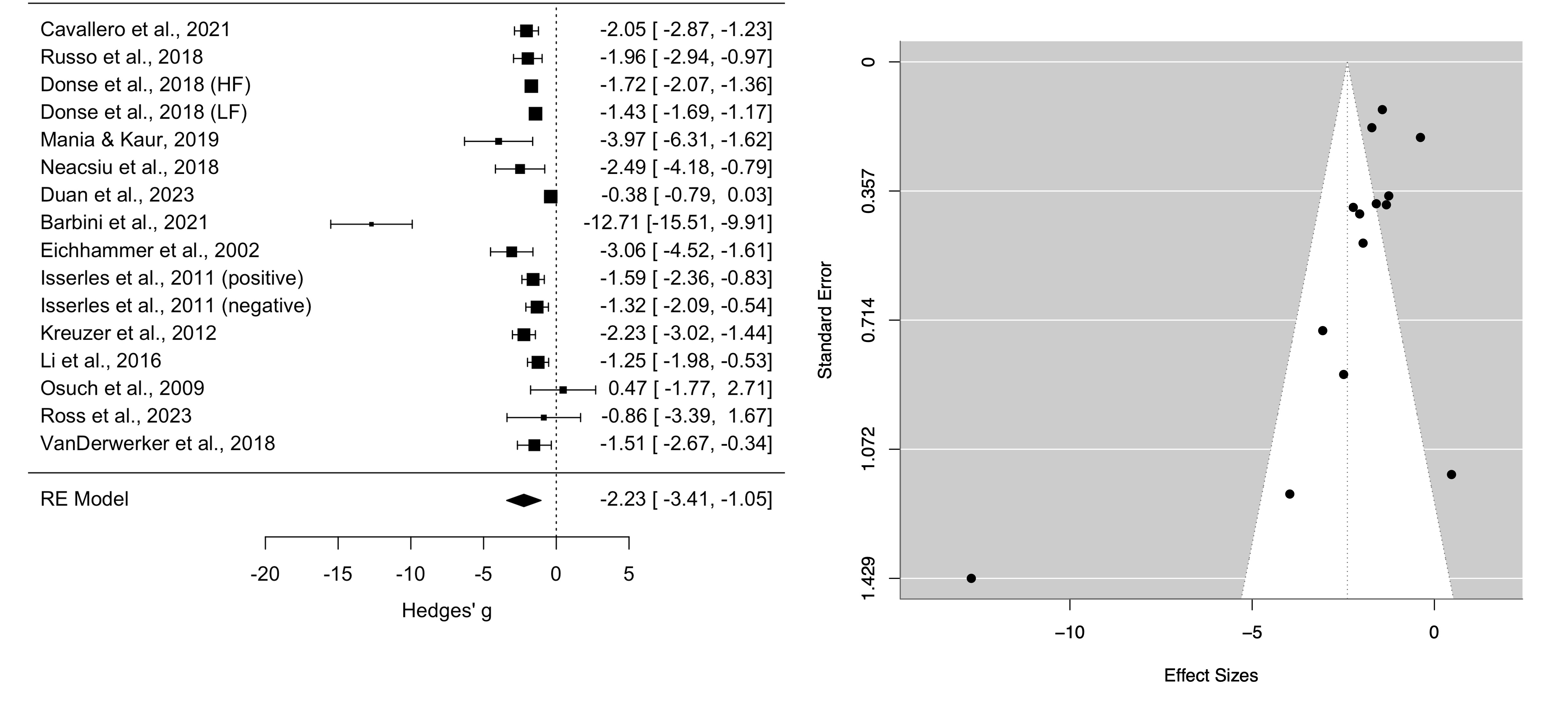

Supplement: Giron et al. supplementary material [file S0033291725000315sup001.zip › Supplementary Figure 2.jpg]
